# The Impact of a Gameful Breathing Training Visualization on Intrinsic Experiential Value, Perceived Effectiveness, and Engagement Intentions: Between-Subject Online Experiment

**DOI:** 10.2196/22803

**Published:** 2021-09-14

**Authors:** Yanick Xavier Lukic, Shari Shirin Klein, Victoria Brügger, Olivia Clare Keller, Elgar Fleisch, Tobias Kowatsch

**Affiliations:** 1 Centre for Digital Health Interventions Department of Management, Technology, and Economics ETH Zurich Zurich Switzerland; 2 Centre for Digital Health Interventions Institute of Technology Management University of St. Gallen St. Gallen Switzerland

**Keywords:** breathing training, serious game, digital health, mobile health, mHealth, mobile phone, experiential value, instrumental value, online experiment

## Abstract

**Background:**

Slow-paced breathing has been shown to be positively associated with psychological and physiological health. In practice, however, there is little long-term engagement with breathing training, as shown by the usage statistics of breathing training apps. New research suggests that gameful smartphone-delivered breathing training may address this challenge.

**Objective:**

This study assesses the impact of breathing training, guided by a gameful visualization, on perceived experiential and instrumental values and the intention to engage in such training.

**Methods:**

A between-subject online experiment with 170 participants was conducted, and one-way multiple analysis of variance and two-tailed *t* test analyses were used to test for any difference in intrinsic experiential value, perceived effectiveness, and the intention to engage in either a breathing training with a gameful or a nongameful guidance visualization. Moreover, prior experience in gaming and meditation practices were assessed as moderator variables for a preliminary analysis.

**Results:**

The intrinsic experiential value for the gameful visualization was found to be significantly higher compared to the nongameful visualization (*P*=.001), but there was no difference in either perceived effectiveness (*P*=.50) or the intention to engage (*P*=.44). The preliminary analysis of the influence of meditation and gaming experience on the outcomes indicates that people with more meditation experience yielded higher intrinsic experiential values from using the gameful visualization than people with no or little meditation experience (*P*=.03). This analysis did not find any additional evidence of gaming time or meditation experience impacting the outcomes.

**Conclusions:**

The gameful visualization was found to increase the intrinsic experiential value of the breathing training without decreasing the perceived effectiveness. However, there were no differences in intentions to engage in both breathing training conditions. Furthermore, gaming and meditation experiences seem to have no or only a small positive moderating effect on the relationship between the gameful visualization and the intrinsic experiential value. Future longitudinal field studies are required to assess the impact of gameful breathing training on actual behavior, that is, long-term engagement and outcomes.

## Introduction

### Background

Slow-paced breathing training has been positively associated with psychological and physiological well-being. It has the potential to help ease the burden of mental illnesses [1] and chronic diseases [2], such as hypertension [3,4], type 2 diabetes [5], and chronic pain [6]. Slow-paced breathing has been shown to influence heart rate variability and cardiac vagal tone positively. The latter corresponds to the activity of the parasympathetic nervous system [7]. Therefore, often the main goal of slow-paced breathing training is to induce relaxation.

The use of smartphones and apps is increasing as they support users’ daily activities [8,9]. For example, smartphone apps can support health and medical actions [9]. Moreover, persuasive technologies and digital interventions are showing promise in influencing personal and health behavior changes [10,11].

This increase in the use of digital technologies and the positive effects of breathing training have led to the development of various applications that support breathing training [12-14]. Usually, such breathing training applications consist of an animation that guides users to breathe at a rate of 6 breaths per minute. The users usually breathe following a specific breathing pattern; for example, 5 seconds inhalation and 5 seconds exhalation [15].

While adapting to such healthy behaviors can help prevent the onset of many diseases [16], behavior changes are challenging to sustain long-term [17]. It has been shown that breathing training apps have very low long-term adherence, even though the number of installations is high [18].

One approach to addressing low long-term adherence might be to approach intrinsic motivation through gamification [19]. Gamification adds game elements to nongame tasks to make them more engaging and enjoyable. This approach is motivated by the notion that playing a game is generally considered an enjoyable experience [20]. Therefore, the combination of technology to influence human behavior [21] and gamification in the health care context [22] offers a promising benefit for people, especially regarding health behavior change and long-term usage.

It is thus not surprising that experiential values, such as fulfillment, enjoyment, meaningfulness, or playfulness, have received growing recognition in recent years [23,24]. Liu et al [25] introduced the term meaningful engagement as the main goal of gamification. It constitutes the experiential and instrumental values of a gamified system. In terms of breathing training, the instrumental value is the effectiveness of the training in inducing the desired effects, such as relaxation. Meaningful engagement can be the deciding factor for the continued use of a system [26]. However, it must be ensured that an increase in experiential values does not reduce the effects regarding relaxation [25]. For example, a previous study [19] collected breathing rate recordings and heart rate variability-derived measures to ensure that gameful breathing training does not impair the effectiveness of breathing training. While the study found that gameful breathing training is as effective as normal breathing training, it did not investigate whether there was an experiential gain in gameful breathing training. Chittaro et al [12] researched the effectiveness of different nongame breathing training guidance based on visualizations and audio. The authors found that some types of guidance are more effective than others. However, they did not investigate gameful visualizations, and they did not report the experiential values of these different visualizations. Furthermore, whether participants would have the intention to engage in such breathing training in their everyday life, guided by these different visualizations, remains unknown.

### Objectives

In this study, we compare gameful and standard slow-paced breathing training visualizations regarding the intrinsic dimension of the experiential value. This perceived intrinsic value serves as a concept to evaluate the effectiveness of the visual components of the gameful design. As a basis for the gameful breathing training, we used Breeze (Centre for Digital Health Interventions), which was first introduced by Shih et al [27]. Additionally, perceived effectiveness was measured to ensure that the gameful design elements do not weaken the instrumental aspect of the task. Furthermore, we measured the intention to engage, that is, the intention to perform the breathing training regularly. Intention to engage was used as a surrogate to study potential long-term engagement.

Moreover, the study investigated the impact of gaming time and meditation experience on the main outcome, namely the intrinsic dimension of the experiential value. However, the analysis was not powered for this latter analysis.

Consequently, this study aimed to compare gameful and nongameful breathing training visualizations regarding different perceived aspects and thus pursued the following objectives:

1. To compare intrinsic values.
2. To compare perceived effectiveness.
3. To compare the intention to engage as a preliminary forecast of long-term engagement.
4. To preliminarily assess the influence of gaming time and meditation experience on the intrinsic value, perceived effectiveness, and intention to engage.

## Methods

### Study Design

**Figure 1:**
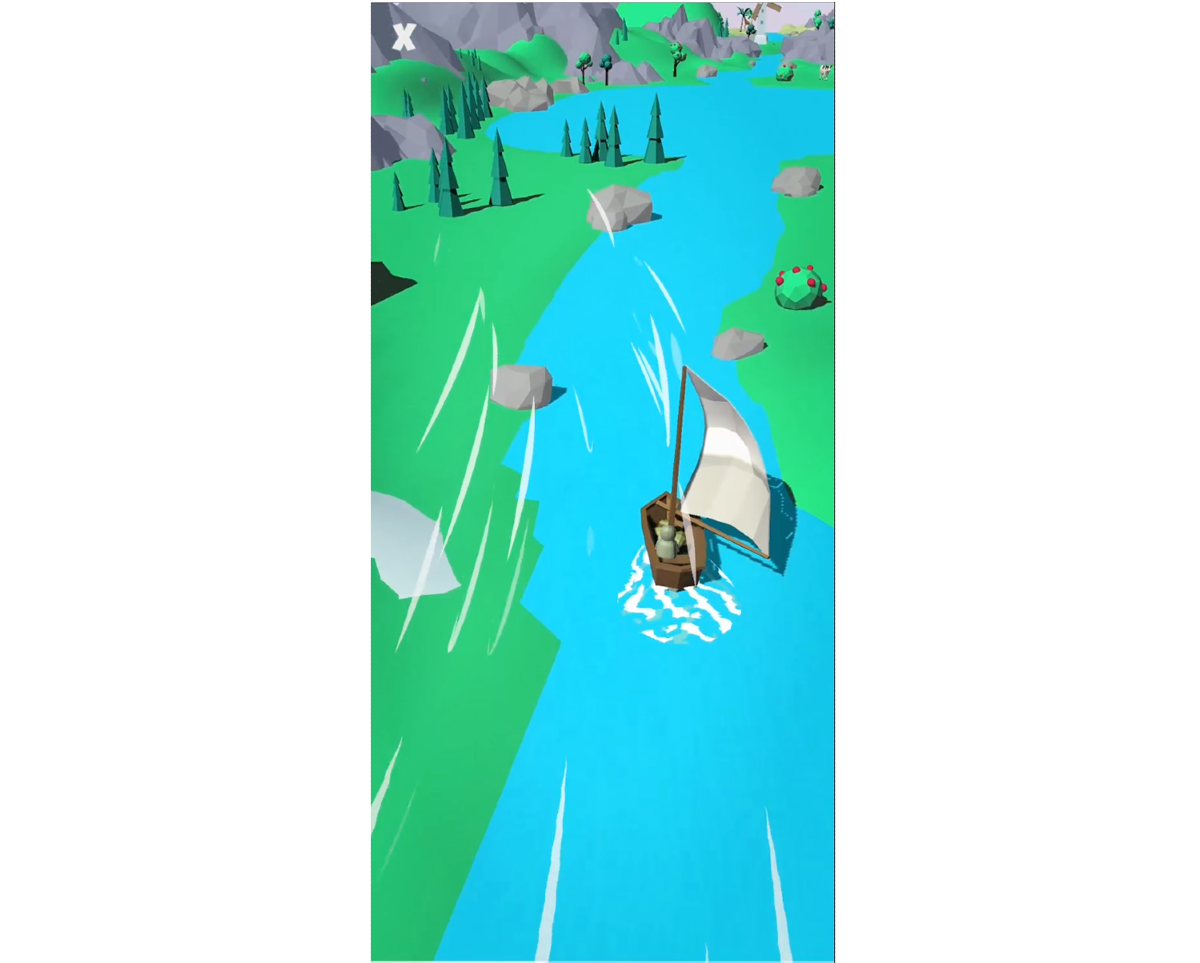
Breeze, the developed gameful breathing training visualization.

**Figure 2:**
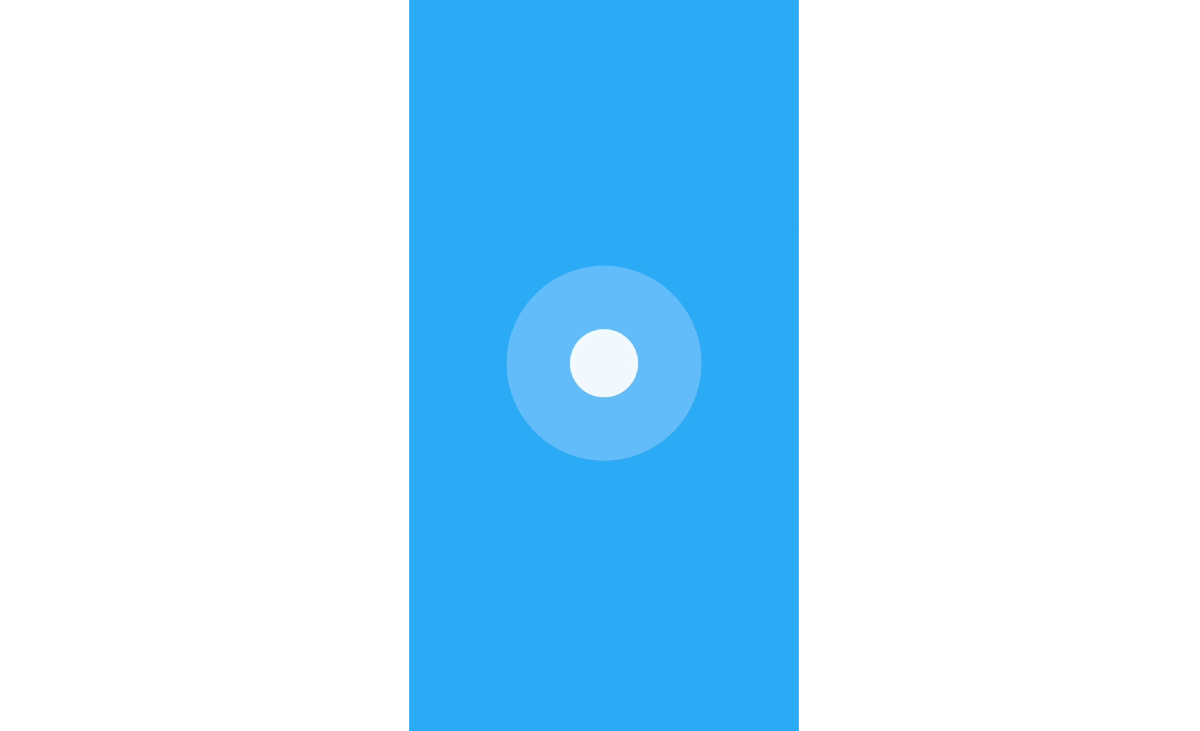
Circle, the standard breathing training visualization.

The study compared Breeze (Figure 1), a gameful breathing training visualization, to a standard breathing training visualization, hereafter referred to as Circle (Figure 2). The standard breathing training visualization was adapted from previous studies [15,19,27]. The videos used to instruct and guide the participants before and during breathing training can be found in Multimedia Appendices 1-4. Both breathing training visualizations guide the user to breathe at a rate of six breathing cycles per minute. We employed a 4-3-3 (inhalation-exhalation-pause) pattern in both visualizations. It is a slight variation from the 4-2-4 breathing pattern from Russel et al [15]. The increase of the exhalation phase was motivated by feedback from testers that felt that the exhalation phase was too short.

The anonymized study was conducted from June 2020 to July 2020 and followed a between-subject design amid the independent variable visualization versions, Breeze or Circle. The study was reviewed and approved by the ethics committee of the Swiss Federal Institute of Technology Zurich (ID: 2020-N-82).

### Main Outcomes

The intrinsic dimension of the experiential value induced by Breeze and Circle formed the major outcome of the study. The intrinsic dimension consists of the subdimensions’ aesthetics and playfulness. To measure the intrinsic experiential value, referred to as intrinsic value in this study, we adapted items from the validated value landscape suggested by Mathwick et al [28] that have also been used in the mobile gaming context [29]. However, solely the intrinsic value was included, as the extrinsic values, consumer return on investment, and service excellence are made for goods on the market. Consequently, no price and no sales processes are in place for Breeze or Circle, nor do they include a service component.

Liu et al [25] suggested that meaningful engagement consists of experiential values and instrumental values. An earlier study [19] had collected breathing rate recording and heart rate variability-derived measures to ensure that the instrumental values are maintained when using Breeze. Therefore, this study focused mainly on the second category—the experiential value to evaluate experiential outcomes.

Besides the experiential value, the instrumental value, consisting of perceived effectiveness (including perceived relaxation) suggested by Chittaro et al [12], was investigated to ensure that the desired effects are achieved. Moreover, intention to use, referred to as intention to engage in this study, was adopted from Shih et al [27] and included to ensure comparability to this earlier study.

Consequently, the following main hypotheses were formulated:

1. H1: Intrinsic experiential value differs between conditions.
2. H2: Perceived effectiveness does not differ between conditions.
3. H3: Intention to engage differs between conditions.

The study also investigated the effects of the visualizations on the subdimensions’ aesthetics and playfulness of the intrinsic experiential value to gain more insight, resulting in the following hypotheses:

1. H4.1: Aesthetics differs between conditions.
2. H4.2: Playfulness differs between conditions.

The main hypotheses, H1, H2, and H3, were tested using two-tailed independent *t* tests.

A multiple analysis of variance (MANOVA) with subsequent univariate tests was used to test H4.1 and H4.2.

### Explorative Outcome

Additionally, an exploratory analysis was carried out regarding the differences in reported intrinsic experiential values, perceived effectiveness, and intention to engage between participants with different levels of experience in gaming and meditation. On the one hand, affluent gaming experiences are suspected to diminish the effectiveness of gamification [30]. On the other hand, mindfulness meditation was suggested to promote health and performance [31,32]. Both these conjectures could influence the experiential outcome.

The effects on the intrinsic value, perceived effectiveness, and intention to engage were investigated to investigate whether gaming time or meditation experience impact experiential value, instrumental value, or engagement. This was done using pairwise independent *t* tests on subgroups in both conditions. For the analysis of gaming experience, the subgroups were gamers and nongamers, and for meditation experience, meditation experts and nonexperts.

### Questionnaire

The questionnaire was structured for both conditions (Circle and Breeze) as follows:

1. Demographic questions.
2. Instruction video with questions regarding the intrinsic value, perceived effectiveness, and intention to engage.

The demographic questionnaire asked participants about, amongst other things, their age, gender, and their experience with relaxation and meditation techniques (Multimedia Appendix 5).

A questionnaire with the items listed in Table 1 was employed to measure the participants’ intrinsic value, perceived effectiveness, and intention to engage. Each item was rated on a 5-point Likert scale (1=strongly disagree and 5=strongly agree). Items I-IV were averaged to assess the intrinsic value, consisting of playfulness (items I-II) and aesthetics (items III-IV), and items V-X were averaged to assess the perceived effectiveness of both conditions.

The items used in the questionnaire were based on existing questionnaires, items, and keywords [12,28].

**Table 1:** Scale item wordings of each construct (intrinsic value, perceived effectiveness, and intention to engage).^a^

Construct			Scale item wording		Cronbach *α*		
				Circle		Breeze	
**Intrinsic value**					.72		.62
	I	Doing the breathing training makes me feel I am in another world.					
	II	I enjoy doing this breathing training.					
	III	I like the way the breathing training looks.					
	IV	I think the breathing training is very entertaining.					
**Perceived effectiveness**					.78		.72
	V	The breathing training facilitates relaxation.					
	VI	The breathing training is pleasant to use.					
	VII	It is easy to follow the breathing training instructions.					
	VIII	The breathing training effectively teaches how to breath.					
	IX	The breathing training is effective in reducing stress.					
	X	The breathing training is effective in increasing attention to breath.					
**Intention to engage**					N/A^b^		N/A
	XI	I would perform this breathing training in my everyday life to better manage stressful situations.					

^a^Cronbach *α* of each construct and both conditions Circle and Breeze are described to measure the internal consistency.

^b^N/A: Not applicable, as the intention to engage only consists out of one item.

### Sample Size

Prior to the study, a power analysis was conducted to determine the required sample size. A MANOVA with two groups and two response variables was chosen as the basis of the power analysis to be able to also draw conclusions about the two subdimensions of intrinsic value (playfulness and aesthetics). The parameters for the power analysis were a significance level ( error probability) of .05, an effect size of 0.0625, and a power (1- error probability) of .80, suggesting a sample size of n=158. The participant sample size exceeded the determined sample size due to the inclusion of a safety margin. Thus, the study aimed to recruit 170 participants.

### Participants

The study was conducted in two phases. First, a pilot study with 8 participants (all females) was carried out to eliminate ambiguities and verify the technical setup. Second, the main study was carried out with 170 participants (85 females). The participants were recruited from the web-based prolific.co platform [33].

Participants were eligible for the study if they were at least 18 years old, were not pregnant, were not taking any medication to treat symptoms of depression, anxiety, or low mood, and were not suffering from any respiratory diseases, such as asthma or chronic obstructive pulmonary disease. The participants were filtered by the platform to match the selection and exclusion criteria. Moreover, participants were expected to take approximately 15 minutes for the questionnaire. After completing the experiment, the participants received compensation worth £2.09 (US $2.68).

### Procedure

**Figure 3:**
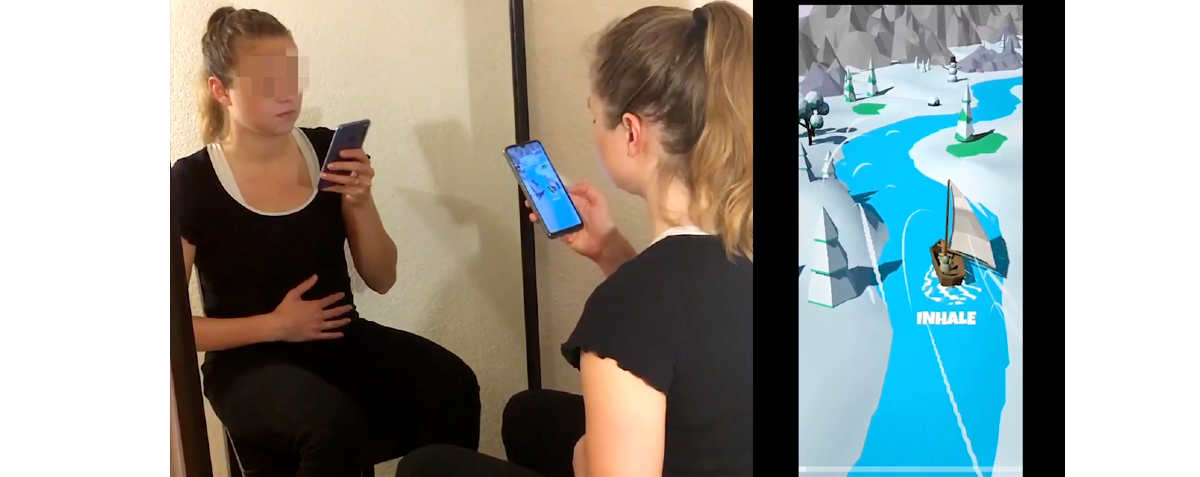
Exercise instruction: Person on the left showing the exercise with enlarged smartphone screen of Breeze on the right. Participants in the Circle condition were shown a similar instruction video with Circle.

Participants were greeted through an explanatory introduction text informing them that they would conduct a breathing exercise and subsequently answer several assessment questions. After accepting the terms and conditions, participants were asked to answer a set of demographic questions. The participants were then randomly assigned to the Circle or the Breeze condition. Subsequently, they were shown an instruction video (Figure 3), accompanied by a tutorial. Next, they were asked to confirm that the breathing training would be taken seriously to convey a binding character. In addition, the participants were asked to follow the guided breathing instructions for 6 minutes delivered through a video of Circle or Breeze, respectively. During this time, the participants were unable to rewind or fast-forward the video. Also, the survey could not be continued before the full 6 minutes of the video had elapsed. Upon completion, the participants were asked about the perceived effects of the breathing training and how they perceived the visualization. Finally, they were led to the final debriefing view, where the purpose of this study was explained to them. For the complete questionnaire or tutorial, see Multimedia Appendix 5.

## Results

All analyses were performed in Rstudio (version 1.2.5042; Rstudio and PBS) and R (version 4.0.0; R Core Team). For constructs that consisted of multiple items, the average of the items was calculated to arrive at a single score for each construct. All measures met assumptions of normality, and no transformations were made.

### Participant Demographics

The study was attempted by 180 participants, of which 10 did not complete the experiment, either because they abandoned the experiment or exceeded the time limit of the prolific.co platform [33]. The platform automatically replaced these failed attempts, resulting in 170 participants completing the experiment. Of these participants, 14 participants were excluded from subsequent analysis due to requiring more than 10 minutes for the breathing training. This threshold was set to account for participants who likely did not take the training seriously, as they stayed on the training page for a long time after the 6 minutes of training. This resulted in a sample size of N=156. The participants’ mean age was 27 years (SD 8.47). Additional data on participant demographics are depicted in Table 2.

**Table 2:** Study participant demographics and mean responses.

Variable		Overall responses (N=156)
**Visualization, n (%)**		
	Breeze	79 (50.6)
	Circle	77 (49.4)
**Sex, n (%)**		
	Women	77 (49.4)
	Men	79 (50.6)
Age, mean (SD)		27.00 (8.47)
**Gaming time, n (%)**		
	Never	27 (17.3)
	1-3 hours	52 (33.3)
	4-6 hours	28 (17.9)
	7-9 hours	22 (14.1)
	10-12 hours	10 (6.4)
	13+ hours	17 (10.9)
**Meditation experience, n (%)**		
	Strongly disagree	34 (21.8)
	Disagree	53 (34.0)
	Neither agree nor disagree	38 (24.4)
	Agree	29 (18.6)
	Strongly agree	2 (1.3)
**Breathing experience, n (%)**		
	Strongly disagree	34 (21.8)
	Disagree	53 (34.0)
	Neither agree nor disagree	38 (24.4)
	Agree	29 (18.6)
	Strongly agree	2 (1.3)
Intrinsic value, mean (SD)		3.30 (0.68)
Perceived effectiveness, mean (SD)		4.07 (0.54)
Intention to engage, means (SD)		3.48 (1.04)

### Intrinsic Value, Perceived Effectiveness, and Intention to Engage

The questionnaire responses regarding the intrinsic value and perceived effectiveness were evaluated by factor analysis [12,34], as the intention to engage only consisted of one item. For the analyzed data sets and conditions, the Kaiser-Meyer-Olkin was greater than 0.60, and Bartlett’s Test of Sphericity was significant (Table 3). Therefore, factor analysis could be conducted [12,35] (Multimedia Appendix 6).

**Table 3:** Barlett’s Test of Sphericity observed on questionnaire data.

	KMO^a^		*P* value	
	Circle	Breeze	Circle	Breeze
Intrinsic value	0.71	0.60	<.001	<.001
Perceived effectiveness	0.79	0.73	<.001	<.001
Intention to engage	N/A^b^	N/A	N/A	N/A

^a^KMO: Kaiser-Meyere-Olkin.

^b^N/A: Not applicable, as the intention to engage only consists of one item.

A pairwise independent *t* test was used to investigate the effects of the conditions on intrinsic value, perceived effectiveness, and intention to engage. A conservative alpha level of .025 was used to reduce type I errors [36].

**Figure 4:**
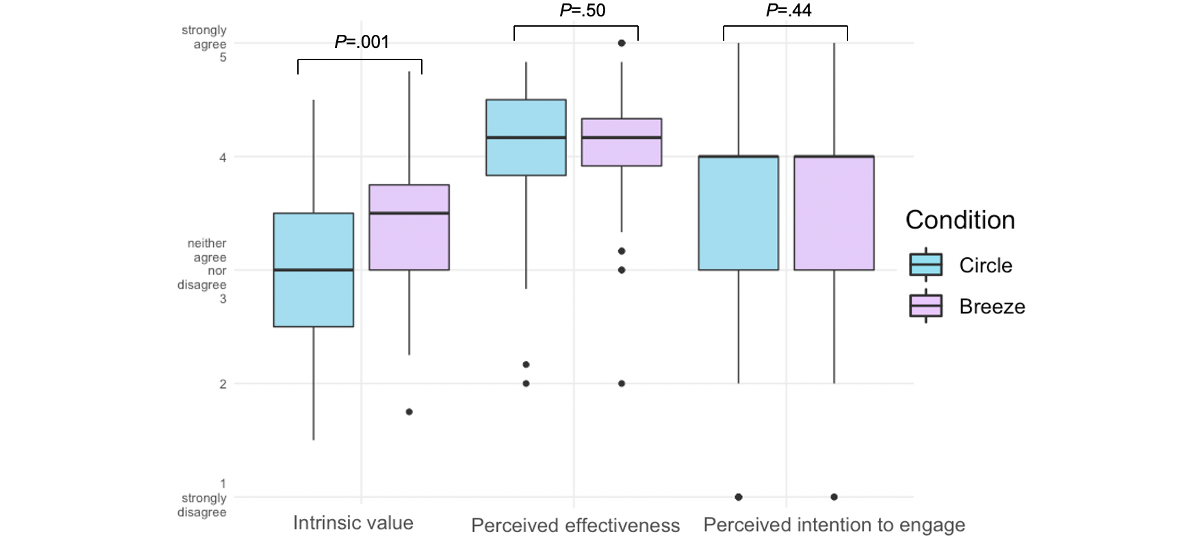
Boxplots, representing intrinsic value, perceived effectiveness and intention to engage median of both conditions (Circle and Breeze). Error bars indicate standard error of the median.

The comparison revealed a significant difference between Breeze and Circle for intrinsic value (*P*=.001), with a positive effect of Breeze (Figure 4). However, there was no significant difference between perceived effectiveness and intention to engage (Table 4). The analysis yields a medium effect size (Cohen’s *d*) for the intrinsic value and a small effect size for perceived effectiveness and intention to engage [37,38] (Table 4).

**Table 4:** Items of intrinsic value, perceived effectiveness, and intention to engage were averaged of both conditions (Circle and Breeze) to form a reliable scale.

	Circle, mean (SD)	Breeze, mean (SD)	*t* test (*df*)	*P* value	Cohen’s *d* (95% CI)
Intrinsic value	3.12 (0.73)	3.47 (0.58)	–3.34 (145)	.001	0.54 (0.21 to 0.86)
Perceived effectiveness	4.04 (0.57)	4.09 (0.50)	–0.67 (150)	.50	0.11 (–0.21 to 0.42)
Intention to engage	3.42 (1.08)	3.54 (1.01)	–0.77 (153)	.44	0.12 (–0.19 to 0.44)

### Differences in Subdimensions Playfulness and Aesthetics

MANOVA was performed to investigate the condition differences of the subdimensions of intrinsic values (playfulness and aesthetics). A conservative alpha level was applied to reduce the risk of type I errors, as suggested by Cohen [36] and Chittaro et al [12]. Thus, a Bonferroni corrected conservative alpha level of .0125 was used, which was assessed by dividing the anticipated alpha level of .05 for the MANOVA by 4, the number of dependent variables included in the MANOVA.

The one-way MANOVA showed a statistically significant difference between the two conditions on the combined dependent variables aesthetics and playfulness (Wilks’ *λ*=0.915, *F*_2, 153_=7.083; *P*=.001; partial *η*^2^=0.085).

Subsequent univariate tests (with Bonferroni correction) yielded a significant difference for the aesthetics dimension (*P*<.001) between conditions, with Breeze yielding a higher value. For playfulness, the difference was also higher for the Breeze condition. The difference, however, was not significant (*P*=.041).

**Table 5:** Subsequent univariate tests for the MANOVA on the condition differences for the subdimensions aesthetics and playfulness of the intrinsic value.

	Circle, mean (SD)	Breeze, mean (SD)	*F* test (*df*)	*P* value
Aesthetics	3.20 (0.80)	3.64 (0.67)	14.20 (154)	<.001
Playfulness	3.05 (0.82)	3.30 (0.74)	4.25 (154)	.041

### Influence of Gaming Time and Meditation Experience

Furthermore, explorative analysis of the influence of gaming time and meditation experience on the intrinsic value, perceived effectiveness, and intention to engage was conducted. The median of gaming time (>3 h/week) was used to assign participants into gamers (>3 h/week) and nongamers (≤3 h/week). Furthermore, participants were classified as meditation experts if they responded with “agree” or “strongly agree” to being experienced in meditation. The mean intrinsic value for gamers was slightly higher in both conditions, yet not significant (mean 3.18, SD 0.65 vs mean 3.06, SD 0.80 for nongamers; *P*=.50) for Circle and (mean 3.51, SD 0.60 vs mean 3.44, SD 0.56 for nongamers; *P*=.60) for Breeze. For perceived effectiveness, there was no difference between gamers and nongamers for Circle (mean 4.06, SD 0.61 vs mean 4.01, SD 0.54, respectively; *P*=.77) and Breeze (mean 4.09, SD 0.58 vs mean 4.10, SD 0.42, respectively; *P*=.87. For the intention to engage, there was no significant difference between gamers and nongamers for Circle (mean 3.47, SD 1.01 vs mean 3.36, SD 1.16, respectively; *P*=.64) and Breeze (mean 3.62, SD 1.07 vs mean 3.48, SD 0.96, respectively; *P*=.52). The Circle condition had similar mean intrinsic values of mean 3.13 (SD 0.71) for meditation experts and mean 3.13 (SD 0.74) for nonmeditation experts (*P*=.98), and the Breeze condition’s mean intrinsic value significantly decreased (*P*=.03) between experts and nonexperts (mean 3.78, SD 0.52 vs mean 3.41, SD 0.57, respectively). For perceived effectiveness, there was no significant difference. Circle had a mean of 4.17 (SD 0.46) for meditation experts compared to a mean of 4.01 (SD 0.59) for nonmeditation experts (*P*=.21), and Breeze had a mean of 4.11 (SD 0.76) for meditation experts compared to a mean of 4.09 (SD 0.44) for nonmeditation experts (*P*=.94). Intention to engage showed a slight decrease for nonmeditation experts (mean 3.37, SD 1.10 for Circle and mean 3.49, SD 1.03 for Breeze) compared to meditation experts (mean 3.64, SD 1.01 for Circle and mean 3.79, SD 0.89 for Breeze). However, for both Circle (*P*=.37) and Breeze (*P*=.29), the differences were not significant. Additionally, there seemed to be no moderating effect of gaming time (>3 h/week) or meditation expertise (“agree” and “strongly agree”) on the relationship of intrinsic value, perceived effectiveness, and intention to engage with the condition (Multimedia Appendix 7).

## Discussion

### Intrinsic Experiential Value and Intention to Engage Feedback

The intrinsic value for the Breeze visualization was higher than the standard breathing training visualization Circle. Therefore, the gameful visualization was well received by the respondents and successfully found to raise the intrinsic value. The analysis of the subdimensions of intrinsic value, aesthetics, and playfulness showed that both subdimensions scored higher for Breeze. The dimension playfulness, however, did not increase significantly. It could be argued that this may be because the video-delivered gameful breathing training visualization did not incorporate an interactive component. It is plausible that an interactive component of Breeze (eg, stronger acceleration of the boat when exhaling at the right time) might further elevate both subdimensions and the intrinsic dimension of the experiential value as a whole. However, a stronger influence on playfulness than aesthetics would be expected since the aesthetics dimension is less strongly linked to interactive aspects. Nevertheless, further research is needed to confirm this.

In addition to higher scores in intrinsic value and its subdimensions, it was hypothesized that the Breeze condition would be associated with a higher intention to engage. This would have been an additional confirmation that using a gameful visualization in breathing training would potentially be beneficial for long-term engagement. However, the hypothesis could not be confirmed as no difference in intention to engage was observed between the two conditions. Nevertheless, these results are comparable to the results of a previous study [27], in which a small number of participants engaged in both gameful and nongameful breathing training. That study also found only nonsignificant increased values for intention to engage for gameful breathing training.

Consequently, it can be concluded that while a gameful visualization component results in a higher intrinsic value, it appears not to increase the behavioral intention to engage with breathing training significantly. At this time, it remains unclear whether a gameful visualization component could help improve long-term engagement.

### Perceived Effectiveness

The participants reported a clearly positive perceived effectiveness of the training. Furthermore, no significant differences were found regarding perceived effectiveness among the Breeze and Circle condition. Thus, the gameful visualization of Breeze had no impairing impact on the instrumental value of the breathing training, which is crucial as gamification should not reduce the instrumental value, even if the experiential value increases [19,25]. These results align with our hypotheses and confirm results from previous work where the influences of gameful and nongameful breathing training on the physiological outcomes were investigated [27].

### Gaming and Meditation

No influence of gaming time per week (≤3h or >3h) was observed among conditions, as implied by the lack of significant differences in mean intrinsic value, perceived effectiveness, and intention to engage. Also, no effect was found for meditation experience on perceived effectiveness and intention to engage in both conditions. However, a significant difference (*P*=.03) between meditation experts and nonexperts was found for intrinsic value in the Breeze condition. Hence, the gameful visualization was better received by meditation experts, but for the Circle condition, no differences were found between the meditation subgroups. Nevertheless, the moderating effect of both gaming time and meditation did not influence the relationship between the conditions regarding the intrinsic value, perceived effectiveness, or intention to engage.

### Limitations

This study was conducted with the users’ feedback via video; therefore, it did not contain biofeedback similar to previous studies employing Breeze [19,27]. Biofeedback is essential to provide individuals with real-time feedback information so they can adjust their behavior by modifying their physiological functions. As Cugelman [39] suggested, biofeedback is a core ingredient with clear linkage to those proven behavior change strategies. Therefore, it is a crucial part of gameful breathing training, and thus, the inclusion of biofeedback should be further investigated.

Likewise, the evaluation of this study was solely based on perceived indicators and not on physiological measurements. Nevertheless, it can be argued that the online format allowed the exercise to take place in a more realistic setting, as participants were conducting the training in their daily lives rather than in an experimental setting.

The intrinsic dimension of experiential value, composed of playfulness and aesthetics, was chosen as the main outcome because of the overlapping context of the appeal of the design and the emotions induced by the application [28]. However, questionnaire items were selected and slightly adapted to fit with this study. While the reliability analyses yielded scores in the acceptable reliability range [40], the score for intrinsic value in the Breeze condition was on the lower end of the acceptable range. Thus, the results regarding intrinsic value yielded acceptable reliability but should still be regarded with caution. Perceived effectiveness and intention to engage were used similar to previous studies to ensure comparability [12,27]. Moreover, the factor analysis ensured the validity of the items of intrinsic value and perceived effectiveness. However, no factor analysis was conducted for intention to engage, as it only consisted of one item.

One main goal of Breeze is to enhance long-term usage [19]. While this study was able to show an increase in experiential value for gameful breathing training, a long-term study is necessary to conclude whether this increase has a positive effect on long-term usage.

The subgroup analysis of gaming time per week and meditation expertise was secondary data analysis and consequently not powered. Thus, further investigations will be necessary to confirm that gaming time and meditation experience do not have a large impact on the intrinsic value, perceived effectiveness, or intention to engage and whether Breeze is better received by meditation experts. However, this analysis provides a first insight and preliminary results of gaming time and meditation experience influence on the studied dimensions.

Finally, gamification may influence people with different backgrounds in different ways. Thus, cultural and social influences might affect how gamification is best realized regarding breathing training [41]. However, this study did not consider these potential influences since Breeze is not designed with a specific target group in mind, and the participant recruitment process did not include any cultural or social parameters.

### Future Work

As this study was conducted as an online experiment consisting of one session, the conclusions regarding long-term engagement are limited. Therefore, plans exist to test Breeze in a future longitudinal field prototype with various patient populations, including healthy, vulnerable, and sick individuals that require breathing training for different purposes. Future studies might focus on students exhibiting exam stress or depression and cancer and hypertension patients that suffer from distress.

Moreover, a study that includes biofeedback will be crucial to assess whether this technique has an advantageous effect on gamification and consequently on behavior change [39].

Additionally, a collection of physiological data measurements next to the perceived indicators will be beneficial to evaluate the instrumental value of the breathing training. Other concepts, which could be integrated into further research are flow, user engagement, usability, or technology acceptance.

### Conclusions

In this study, we evaluated the difference between a gameful and a nongameful breathing training guidance visualization. The variables measured were intrinsic value representing experiential value, perceived effectiveness representing instrumental value, and intention to engage representing long-term engagement. The results yielded a positive effect by showing that the experiential value of Breeze was higher than the experiential value of Circle without decreasing the instrumental value and the engagement. Subgroup analyses indicated no impact of gaming time (≤3h and >3h per week) and meditation (experts and nonexpert) on intrinsic value, perceived effectiveness, or intention to engage. The only difference identified was that the gameful visualization was better perceived by meditation experts than by nonexperts.

The outcomes showed that the intrinsic dimensions of the experiential value of breathing training could be increased by employing gameful instead of nongameful visualizations without impairing the perceived effectiveness of the training.

## Appendix

Multimedia Appendix 1 Instruction video shown to the participants assigned to the Breeze condition.

Multimedia Appendix 2 Instruction video shown to the participants assigned to the Circle condition.

Multimedia Appendix 3 Training video (6 minutes) shown to the participants assigned to the Breeze condition.

Multimedia Appendix 4 Training video (6 minutes) shown to the participants assigned to the Circle condition.

Multimedia Appendix 5 Employed questionnaire.

Multimedia Appendix 6 Factor analyses for intrinsic experiential value and perceived effectiveness.

Multimedia Appendix 7 Influence of gaming time and meditation experience on the investigated outcomes.

## Abbreviations

MANOVA: multivariate analyses of variance
