# Supplementary material for: The Impact of a Gameful Breathing Training Visualization on Intrinsic Experiential Value, Perceived Effectiveness, and Engagement Intentions: Between-Subject Online Experiment
Source: JMIR Serious Games. 2021 Sep 14;9(3):e22803. doi: 10.2196/22803 (PMC8479602; doi:10.2196/22803)
Supplement: Multimedia Appendix 5 [file games_v9i3e22803_app5.docx]

# Multimedia Appendix 5. Employed questionnaire.

## Questionnaire

**INTRODUCTION**

In this survey, you will be asked to fill out a questionnaire that aims at evaluating a breathing training application. It takes about 15 minutes to complete. After answering general introductory questions, you will be asked to perform a simple breathing task, which is presented by a video. You will then be asked questions about the effect and attractiveness of the exercise and the way it was presented.

**TERMS AND CONDITIONS**

Your responses will be stored strictly anonymous. We do not collect any data that allows us to draw conclusions about your identity. The anonymized data may be published as part of scientific publications and may be published in a publicly available database according to the principles of the open research data initiative of the Swiss National Science Foundation.

**PART 1**

- What is your age? (number input)
- Sex
  - man, woman
- What is the highest level of education you have completed?
  - Secondary Education, Further Academic Education (A-Levels, GNVQ's, BTEC or equivalent), Further Vocational Qualification (BTEC Awards, National Vocational Qualification, City and Guilds Qualification, Apprenticeship or equivalent), Short-cycle tertiary education (e.g. certificates of advanced study programs), Bachelor or equivalent, Master or equivalent, Doctoral or equivalent
- How many hours per week do you play video games on average?
  - Never, 1-3 Hours, 4-6 Hours, 7-9 Hours, 10-12 Hours, 13+ Hours
- I am physically active.
  - 5-point Likert scale: (1) Strongly Disagree, (2) Disagree, (3) Neither Agree nor Disagree, (4) Agree, (5) Strongly Agree)
- I am experienced with meditation.
  - 5-point Likert scale: (1) Strongly Disagree, (2) Disagree, (3) Neither Agree nor Disagree, (4) Agree, (5) Strongly Agree)
- I am experienced with breathing trainings.
  - 5-point Likert scale: (1) Strongly Disagree, (2) Disagree, (3) Neither Agree nor Disagree, (4) Agree, (5) Strongly Agree)
- If >3: Where do you normally conduct such breathing trainings? (free text input)
- Else: else: Where would you conduct such breathing trainings? (free text input)

**PART 2**

- Instruction video, accompanied by the following explanation:
  “The following video will show you how to do the breathing task. You do not have to follow yet. .”
  - 1. Right side: The visualization of the breathing instructions. You will perform a breathing pattern of 4-3-3 (4 seconds breathing in, 3 seconds breathing out, 3 seconds holding breath). The pattern will be repeated over the course of 6 minutes.
    2. Left side: A person performing the exercise in order to demonstrate what you are supposed to do. Please make sure to 1) sit while performing the exercise, 2) keep your back upright, 3) breath in through your nose, breath out through your mouth, 4) try to breathe deeply and calmly into your belly and avoid a pure chest respiration. You may decide to rest your arms on a table in front of you, on your knees, or on your armchairs if this makes it more comfortable for you to keep your back straight.”
- How do you feel?
  - Affective Slider (Pleasure / Arousal)
- Breathing training with video (6 minutes in accordance with), accompanied by the following explanation:
  - *“*You will now do the breathing training for 6 minutes. Please make sure that you are not disturbed and follow the breathing pattern (4-3-3) as demonstrated in the video. You will be able to continue the survey as soon as the video has completed one playthrough. Skipping forward in the video is not possible. We kindly ask you to do the breathing training seriously to provide us with valid scientific data. For the best experience, follow the video in fullscreen mode.”
- How do you feel?
  - Affective Slider (Pleasure / Arousal)
- Perceived effectiveness (all 5-point Likert scales: (1) Strongly Disagree, (2) Disagree, (3) Neither Agree nor Disagree, (4) Agree, (5) Strongly Agree)
  - The breathing training facilitates relaxation.
  - The breathing training is pleasant to use.
  - It is easy to follow the breathing training instructions.
  - The breathing training effectively teaches how to breath.
  - The breathing training is effective in reducing stress.
  - The breathing training is effective in increasing attention to breath.
- Aesthetics (all 5-point Likert scales: (1) Strongly Disagree, (2) Disagree, (3) Neither Agree nor Disagree, (4) Agree, (5) Strongly Agree)
  - I like the way the breathing training looks. (visual appeal)
  - I think the breathing training is very entertaining. (entertainment value)
- Playfulness (all 5-point Likert scales: (1) Strongly Disagree, (2) Disagree, (3) Neither Agree nor Disagree, (4) Agree, (5) Strongly Agree)
  - Doing the breathing training makes me feel I am in another world. (escapism)
  - I enjoy doing this breathing training. (enjoyment)
- Behavioral intention to use (5-point Likert scale: (1) Strongly Disagree, (2) Disagree, (3) Neither Agree nor Disagree, (4) Agree, (5) Strongly Agree)
  - I would perform this breathing training in my everyday life to better manage stressful situations.

**DEBRIEFING**

With this survey we aim to compare the visualizations of a gamified breathing training to a standard breathing training. Which version you received was random. Also, we hope to receive some feedback for potential future development directions for the gamified breathing training. If you have any questions about this study feel free to contact us: ylukic@ethz.ch
