# Supplementary material for: The Impact of a Gameful Breathing Training Visualization on Intrinsic Experiential Value, Perceived Effectiveness, and Engagement Intentions: Between-Subject Online Experiment
Source: JMIR Serious Games. 2021 Sep 14;9(3):e22803. doi: 10.2196/22803 (PMC8479602; doi:10.2196/22803)
Supplement: Multimedia Appendix 6 [file games_v9i3e22803_app6.docx]

# Multimedia Appendix 6 – Factor analyses for intrinsic experiential value and perceived effectiveness.

The factor analyses were conducted using *jamovi 1.6.23.0* [1]. Since this study is concerned with drawing conclusions within the collected sample, the main factor analysis approach used was principal component analysis (PCA) [2]. To give an indication of the generalizability of the study’s results to other populations, also the exploratory factor analyses (EFA) using the maximum likelihood extraction method are provided for intrinsic experiential value and perceived effectiveness. However, the sample size per condition may not be sufficient to yield adequate results [2] since an evaluation of construct validity is not an objective of this study and it builds upon validated constructs. Nevertheless, the analyses are conducted for each condition (Breeze and Circle) separately to check factorial validity in both conditions.

The number of present factors/components were determined using a combination of parallel analysis, Kaiser’s criterion, and visual inspection of Scree plots.

## Intrinsic Experiential Value

Intrinsic experiential value is made up of four items where items I and II (*escapism*, *enjoy*) represent *playfulness* and items III and IV (*visual appeal*, *entertain*) represent *aesthetics*.

Since all items should measure one factor (*intrinsic experiential value*) the factor analyses were expected to yield one factor where all items load strongly (>0.4).

### Correlation Matrix

The items of the construct correlate significantly, providing a first indication that they measure related aspects.

| Correlation Matrix *both conditions* | | | | | | | | | |
| --- | --- | --- | --- | --- | --- | --- | --- | --- | --- |
|  |  |  |  |  |  |  |  |  |  |
|  | | **escapism** | | **enjoy** | | **visual appeal** | | **entertain** | |
| escapism |  | — |  |  |  |  |  |  |  |
| enjoy |  | 0.405 | *** | — |  |  |  |  |  |
| visual appeal |  | 0.212 | ** | 0.297 | *** | — |  |  |  |
| entertain |  | 0.410 | *** | 0.487 | *** | 0.373 | *** | — |  |
| Note. * p < .05, ** p < .01, *** p < .001 | | | | | | | | | |
|  | | | | | | | | | |

### Assumption Checks

Both conditions fulfill the preconditions for factor analysis.

| Bartlett's Test of Sphericity | | | | | |
| --- | --- | --- | --- | --- | --- |
|  |  |  |  |  |  |
| **χ²** | | **df** | | **p** | |
| 40.9 |  | 6 |  | < .001 |  |
| Note. *Breeze*. | | | | | |

| KMO Measure of Sampling Adequacy | | | |
| --- | --- | --- | --- |
|  |  |  |  |
|  | | **MSA** | |
| Overall |  | 0.604 |  |
| escapism |  | 0.584 |  |
| enjoy |  | 0.641 |  |
| visual appeal |  | 0.562 |  |
| entertain |  | 0.619 |  |
| Note. *Breeze*. | | | |

| Bartlett's Test of Sphericity | | | | | |
| --- | --- | --- | --- | --- | --- |
|  |  |  |  |  |  |
| **χ²** | | **df** | | **p** | |
| 64.3 |  | 6 |  | < .001 |  |
| Note. *Circle*. | | | | | |

| KMO Measure of Sampling Adequacy | | | |
| --- | --- | --- | --- |
|  |  |  |  |
|  | | **MSA** | |
| Overall |  | 0.710 |  |
| escapism |  | 0.787 |  |
| enjoy |  | 0.676 |  |
| visual appeal |  | 0.827 |  |
| entertain |  | 0.660 |  |
| Note. *Circle*. | | | |

### Principal Component Analysis (PCA)

For both conditions, Kaiser’s criterion suggests the presence of one factor. However, in Breeze the second factor’s eigenvalue is very close to 1.0 and a visual inspection of the scree plot would allow to argue for the inclusion of a second factor. Nevertheless, also the parallel analysis suggests the presence of one factor for both conditions. Consequently, the analysis was conducted for one factor.


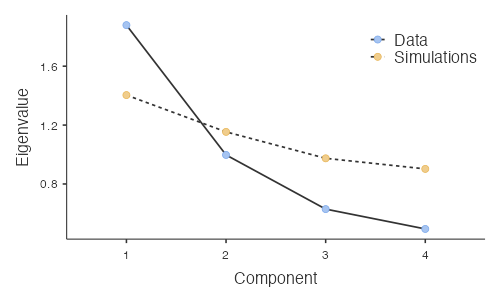


Figure 1. Breeze scree plot (PCA).


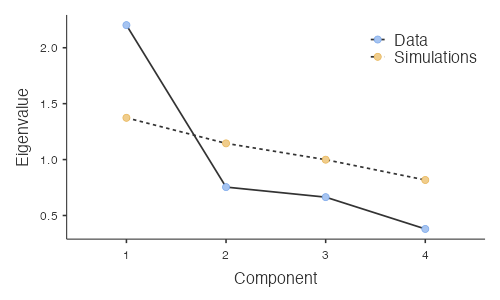


Figure 2. Circle scree plot (PCA).

The component loadings are all above 0.50 in both conditions, indicating that they load quite strongly. The component explains a total of 47.0% and 55.1% of the variance in Breeze and Circle respectively.

| Component Loadings | | | | | |
| --- | --- | --- | --- | --- | --- |
|  | | **Component** | |  | |
|  | | **1** | | **Uniqueness** | |
| escapism |  | 0.692 |  | 0.521 |  |
| enjoy |  | 0.714 |  | 0.490 |  |
| visual appeal |  | 0.573 |  | 0.671 |  |
| entertain |  | 0.749 |  | 0.439 |  |
| Note. *Breeze* | | | | | |
|  | | | | | |

| Component Summary | | | | | | | |
| --- | --- | --- | --- | --- | --- | --- | --- |
|  |  |  |  |  |  |  |  |
| **Component** | | **SS Loadings** | | **% of Variance** | | **Cumulative %** | |
| 1 |  | 1.88 |  | 47.0 |  | 47.0 |  |
| Note. *Breeze* | | | | | | | |

| Component Loadings | | | | | |
| --- | --- | --- | --- | --- | --- |
|  | | **Component** | |  | |
|  | | **1** | | **Uniqueness** | |
| escapism |  | 0.693 |  | 0.519 |  |
| enjoy |  | 0.807 |  | 0.348 |  |
| visual appeal |  | 0.615 |  | 0.622 |  |
| entertain |  | 0.831 |  | 0.309 |  |
| Note. *Circle* | | | | | |
|  | | | | | |

| Component Summary | | | | | | | |
| --- | --- | --- | --- | --- | --- | --- | --- |
|  |  |  |  |  |  |  |  |
| **Component** | | **SS Loadings** | | **% of Variance** | | **Cumulative %** | |
| 1 |  | 2.20 |  | 55.1 |  | 55.1 |  |
| Note. *Circle* | | | | | | | |

### Exploratory Factor Analysis (EFA)

Kaiser’s criterion suggests the presence of one factor in each condition, which is also supported by the scree plot. However, parallel analysis suggests the presence of a second factor in Breeze. Consequently, the EFA is performed for one and two factors for the Breeze condition to verify that the factors relate to the underlying constructs *playfulness* and *aesthetics*. The EFA is performed using *maximum likelihood* as extraction method in combination with *varimax* rotation.


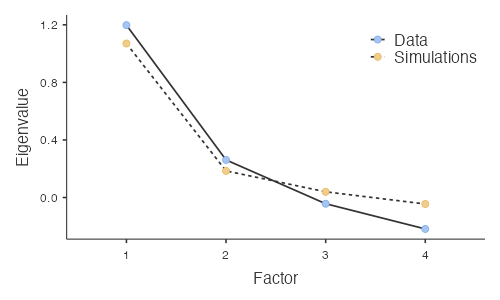


Figure 3. Breeze scree plot (EFA).


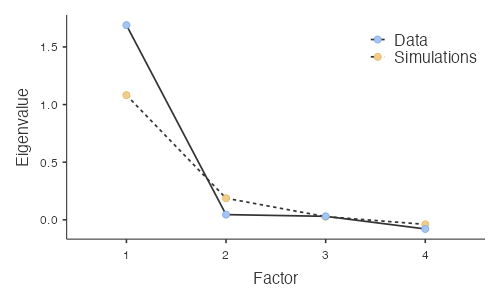


Figure 4. Circle scree plot (EFA).

All factor loadings load quite strongly with above 0.40 in both conditions, except for visual appeal in Breeze which loads with 0.39. Looking at the EFA extracting two factors in Breeze, however, the items separate nicely onto the two factors according to the underlying dimensions playfulness and aesthetics. Only the entertainment value item yields factor loadings of above 0.30 for both factors (0.34). Nevertheless, it loads stronger (0.51) with visual appeal with which it forms the subdimension aesthetics.

The one factor solutions explain a total of 29.0% and 42.1% of the variance in Breeze and Circle respectively. This is rather low, especially in Breeze. The two-factor solution explains a total of 49.3% of the variance, which is more acceptable. Consequently, the one factor may not generalize well outside of the collected sample but should be adequate for this study’s purposes as verified by the PCA [2].

| Factor Loadings | | | | | |
| --- | --- | --- | --- | --- | --- |
|  | | **Factor** | |  | |
|  | | **1** | | **Uniqueness** | |
| escapism |  | 0.585 |  | 0.658 |  |
| enjoy |  | 0.595 |  | 0.646 |  |
| visual appeal |  | 0.390 |  | 0.848 |  |
| entertain |  | 0.591 |  | 0.651 |  |
| Note. *Breeze*, 1 factor. 'Maximum likelihood' extraction method was used. | | | | | |
|  | | | | | |

| Factor Summary | | | | | | | |
| --- | --- | --- | --- | --- | --- | --- | --- |
|  |  |  |  |  |  |  |  |
| **Factor** | | **SS Loadings** | | **% of Variance** | | **Cumulative %** | |
| 1 |  | 1.20 |  | 29.9 |  | 29.9 |  |
| Note. *Breeze*, 1 factor. | | | | | | | |

| Factor Loadings | | | | | | | |
| --- | --- | --- | --- | --- | --- | --- | --- |
|  | | **Factor** | | | |  | |
|  | | **1** | | **2** | | **Uniqueness** | |
| escapism |  | 0.865 |  |  |  | 0.248 |  |
| enjoy |  | 0.485 |  |  |  | 0.711 |  |
| visual appeal |  |  |  | 0.742 |  | 0.446 |  |
| entertain |  | 0.344 |  | 0.509 |  | 0.622 |  |
| Note. *Breeze*, 2 factors. 'Maximum likelihood' extraction method was used in combination with a 'varimax' rotation. Values below 0.30 are omitted. | | | | | | | |
|  | | | | | | | |

| Factor Summary | | | | | | | |
| --- | --- | --- | --- | --- | --- | --- | --- |
|  |  |  |  |  |  |  |  |
| **Factor** | | **SS Loadings** | | **% of Variance** | | **Cumulative %** | |
| 1 |  | 1.105 |  | 27.6 |  | 27.6 |  |
| 2 |  | 0.868 |  | 21.7 |  | 49.3 |  |
| Note. *Breeze*, 2 factors. | | | | | | | |

| Factor Loadings | | | | | |
| --- | --- | --- | --- | --- | --- |
|  | | **Factor** | |  | |
|  | | **1** | | **Uniqueness** | |
| escapism |  | 0.533 |  | 0.716 |  |
| enjoy |  | 0.741 |  | 0.450 |  |
| visual appeal |  | 0.434 |  | 0.812 |  |
| entertain |  | 0.815 |  | 0.336 |  |
| Note. *Circle*. 'Maximum likelihood' extraction method was used. | | | | | |
|  | | | | | |

| Factor Summary | | | | | | | |
| --- | --- | --- | --- | --- | --- | --- | --- |
|  |  |  |  |  |  |  |  |
| **Factor** | | **SS Loadings** | | **% of Variance** | | **Cumulative %** | |
| 1 |  | 1.69 |  | 42.1 |  | 42.1 |  |
| Note. *Circle*. | | | | | | | |

## Perceived Effectiveness

Perceived Effectiveness was introduced and validated by Chittaro et al. Consequently, the factor analysis was conducted to verify that the data set also yields one factor and check the level of explained variance.

### Correlation Matrix

| Correlation Matrix *both conditions* | | | | | | | | | | | | | |
| --- | --- | --- | --- | --- | --- | --- | --- | --- | --- | --- | --- | --- | --- |
|  |  |  |  |  |  |  |  |  |  |  |  |  |  |
|  | | **relaxation** | | **pleasant** | | **ease** | | **teach** | | **stress** | | **attention** | |
| relaxation |  | — |  |  |  |  |  |  |  |  |  |  |  |
| pleasant |  | 0.602 | *** | — |  |  |  |  |  |  |  |  |  |
| ease |  | 0.282 | *** | 0.217 | ** | — |  |  |  |  |  |  |  |
| teach |  | 0.400 | *** | 0.379 | *** | 0.371 | *** | — |  |  |  |  |  |
| stress |  | 0.589 | *** | 0.487 | *** | 0.213 | ** | 0.354 | *** | — |  |  |  |
| attention |  | 0.241 | ** | 0.136 |  | 0.241 | ** | 0.260 | ** | 0.254 | ** | — |  |
| Note. * p < .05, ** p < .01, *** p < .001 | | | | | | | | | | | | | |
|  | | | | | | | | | | | | | |

### Assumption Checks

Both conditions fulfill conditions for factor analysis.

| Bartlett's Test of Sphericity | | | | | |
| --- | --- | --- | --- | --- | --- |
|  |  |  |  |  |  |
| **χ²** | | **df** | | **p** | |
| 96.4 |  | 15 |  | < .001 |  |
| Note. *Breeze.* | | | | | |

| KMO Measure of Sampling Adequacy | | | |
| --- | --- | --- | --- |
|  |  |  |  |
|  | | **MSA** | |
| Overall |  | 0.726 |  |
| relaxation |  | 0.716 |  |
| pleasant |  | 0.659 |  |
| ease |  | 0.795 |  |
| teach |  | 0.770 |  |
| stress |  | 0.828 |  |
| attention |  | 0.589 |  |
| Note. *Breeze.* | | | |

| Bartlett's Test of Sphericity | | | | | |
| --- | --- | --- | --- | --- | --- |
|  |  |  |  |  |  |
| **χ²** | | **df** | | **p** | |
| 128 |  | 15 |  | < .001 |  |
| Note. *Circle*. | | | | | |

| KMO Measure of Sampling Adequacy | | | |
| --- | --- | --- | --- |
|  |  |  |  |
|  | | **MSA** | |
| Overall |  | 0.787 |  |
| relaxation |  | 0.739 |  |
| pleasant |  | 0.830 |  |
| ease |  | 0.756 |  |
| teach |  | 0.820 |  |
| stress |  | 0.781 |  |
| attention |  | 0.838 |  |
| Note. *Circle*. | | | |

### Principle Component Analysis (PCA)

Parallel analysis suggests the presence of one factor for both conditions, which is supported by visual inspection of both scree plots. While Kaiser’s criterion suggests the presence of one factor for the Circle condition, it suggests the presence of two factors in Breeze. Nevertheless, this construct was previously validated, the eigenvalue of the second factor is only marginally larger than 1.0 and the two identification approaches indicate only one factor the PCA was conducted only for one factor for both conditions.


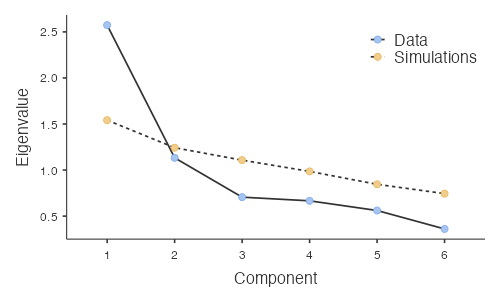


Figure 5. Breeze scree plot (PCA).


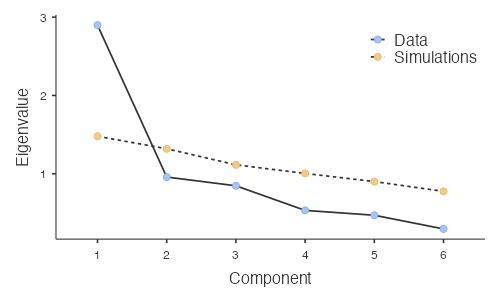


Figure 6. Circle scree plot (PCA).

The component loadings for each of the six items are above 0.40 in both conditions. The component explains a total of 42.9% and 48.3% of the variance in Breeze and Circle.

| Component Loadings | | | | | |
| --- | --- | --- | --- | --- | --- |
|  | | **Component** | |  | |
|  | | **1** | | **Uniqueness** | |
| relaxation |  | 0.789 |  | 0.377 |  |
| pleasant |  | 0.714 |  | 0.490 |  |
| ease |  | 0.591 |  | 0.651 |  |
| teach |  | 0.658 |  | 0.567 |  |
| stress |  | 0.693 |  | 0.519 |  |
| attention |  | 0.422 |  | 0.822 |  |
| Note. *Breeze*. | | | | | |
|  | | | | | |

| Component Summary | | | | | | | |
| --- | --- | --- | --- | --- | --- | --- | --- |
|  |  |  |  |  |  |  |  |
| **Component** | | **SS Loadings** | | **% of Variance** | | **Cumulative %** | |
| 1 |  | 2.57 |  | 42.9 |  | 42.9 |  |
| Note. *Breeze*. | | | | | | | |

| Component Loadings | | | | | |
| --- | --- | --- | --- | --- | --- |
|  | | **Component** | |  | |
|  | | **1** | | **Uniqueness** | |
| relaxation |  | 0.838 |  | 0.298 |  |
| pleasant |  | 0.766 |  | 0.413 |  |
| ease |  | 0.495 |  | 0.755 |  |
| teach |  | 0.707 |  | 0.500 |  |
| stress |  | 0.794 |  | 0.369 |  |
| attention |  | 0.483 |  | 0.767 |  |
| Note. *Circle*. | | | | | |
|  | | | | | |

| Component Summary | | | | | | | |
| --- | --- | --- | --- | --- | --- | --- | --- |
|  |  |  |  |  |  |  |  |
| **Component** | | **SS Loadings** | | **% of Variance** | | **Cumulative %** | |
| 1 |  | 2.90 |  | 48.3 |  | 48.3 |  |
| Note. *Circle*. | | | | | | | |

### Exploratory Factor Analysis (EFA)

Kaiser’s criterion, parallel analysis, and visual inspection of the scree plot all suggested the presence of one factor in both conditions. The EFA is performed using *maximum likelihood* as extraction method.


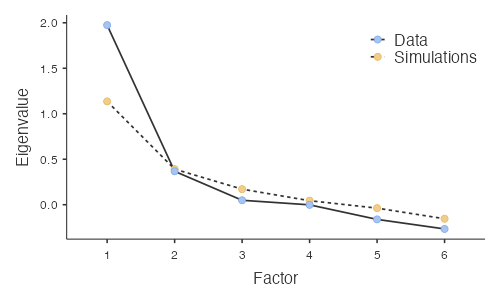


Figure 7. Breeze scree plot (EFA).


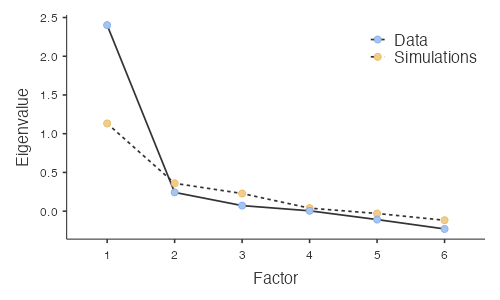


Figure 8. Circle scree plot (EFA).

The factor loadings for each of the six items are above 0.40 in both conditions, except for the *attention* item (0.268 and 0.347 in Breeze and Circle respectively) and the *ease* item (0.347 in Circle). That these items exhibit weaker factor loadings than the other items is consistent with the work that introduced the perceived effectiveness construct [3] even though the factor loadings there were higher.

| Factor Loadings | | | | | |
| --- | --- | --- | --- | --- | --- |
|  | | **Factor** | |  | |
|  | | **1** | | **Uniqueness** | |
| relaxation |  | 0.782 |  | 0.389 |  |
| pleasant |  | 0.691 |  | 0.523 |  |
| ease |  | 0.428 |  | 0.817 |  |
| teach |  | 0.494 |  | 0.756 |  |
| stress |  | 0.611 |  | 0.627 |  |
| attention |  | 0.268 |  | 0.928 |  |
| Note. *Breeze*. 'Maximum likelihood' extraction method was used. | | | | | |
|  | | | | | |

| Summary | | | | | | | |
| --- | --- | --- | --- | --- | --- | --- | --- |
|  |  |  |  |  |  |  |  |
| **Factor** | | **SS Loadings** | | **% of Variance** | | **Cumulative %** | |
| 1 |  | 1.96 |  | 32.7 |  | 32.7 |  |
| Note. *Breeze*. | | | | | | | |

| Factor Loadings | | | | | |
| --- | --- | --- | --- | --- | --- |
|  | | **Factor** | |  | |
|  | | **1** | | **Uniqueness** | |
| relaxation |  | 0.859 |  | 0.261 |  |
| pleasant |  | 0.712 |  | 0.493 |  |
| ease |  | 0.338 |  | 0.886 |  |
| teach |  | 0.567 |  | 0.679 |  |
| stress |  | 0.762 |  | 0.420 |  |
| attention |  | 0.347 |  | 0.880 |  |
| Note. *Circle*. 'Maximum likelihood' extraction method was used. | | | | | |
|  | | | | | |

| Summary | | | | | | | |
| --- | --- | --- | --- | --- | --- | --- | --- |
|  |  |  |  |  |  |  |  |
| **Factor** | | **SS Loadings** | | **% of Variance** | | **Cumulative %** | |
| 1 |  | 2.38 |  | 39.7 |  | 39.7 |  |
| Note. *Circle*. | | | | | | | |

## References

1. The jamovi project. *jamovi*. (Version 1.6) [Computer Software]. In: <https://www.jamovi.org>; 2021.

2. Tinsley HE, Tinsley DJ. Uses of factor analysis in counseling psychology research. *Journal of counseling psychology.* 1987;34(4):414.

3. Chittaro L, Sioni R. Evaluating mobile apps for breathing training: The effectiveness of visualization. *Computers in Human Behavior.* 2014;40:56-63.
