# Supplementary material for: The Impact of a Gameful Breathing Training Visualization on Intrinsic Experiential Value, Perceived Effectiveness, and Engagement Intentions: Between-Subject Online Experiment
Source: JMIR Serious Games. 2021 Sep 14;9(3):e22803. doi: 10.2196/22803 (PMC8479602; doi:10.2196/22803)
Supplement: Multimedia Appendix 7 [file games_v9i3e22803_app7.docx]

# Multimedia Appendix 7. Influence of gaming time and meditation experience on the investigated outcomes.

## Gaming Experience

### Intrinsic Experiential Value

**Table 1.** Mean intrinsic value and standard deviation of lower and upper half of gaming time per week participants, as well as difference between the lower and upper half indicated with t value, degrees of freedom (df), and *P* value.

| Condition | Mean intrinsic value (SD^a^) | | *t* value | df | *P* value |
| --- | --- | --- | --- | --- | --- |
|  |  |  |  |  |  |
|  | G1^b^ | G2^c^ |  |  |  |
| Circle | 3.06 (0.80) | 3.18 (0.65) | -0.68 | 72.9 | .50 |
| Breeze | 3.44 (0.56) | 3.51 (0.60) | -0.53 | 76.37 | .60 |

^a^SD: standard deviation.

^b^G1: participants lower half (median) of gaming time per week.

^c^G2: participants upper half (median) of gaming time per week.

**Table 2.** Moderator analysis of gaming time per week on the relationship of intrinsic value to condition (Breeze or Circle).

| Intrinsic value | Estimate | Std. Error | *t*_(152)_ | *P* value |
| --- | --- | --- | --- | --- |
|  |  |  |  |  |
| Intercept | 3.06 | 0.11 | 29.07 | <.001 |
| Condition^a^ | 0.37 | 0.15 | 2.52 | .01 |
| Gaming G2^b^ | 0.11 | 0.15 | 0.76 | .45 |
| Condition^a^:Gaming G2^b^ | -0.04 | 0.21 | -0.21 | .83 |
| Adjusted R^2^=-0.05431, F_(3, 152)_=3.967, *P* value*=*0.009326 | | | |  |

^a^Conditon: Breeze or standard breathing training (Circle).

^b^Gaming G2: participants upper half (median) of gaming time per week.

### Perceived Effectiveness

**Table 3.** Mean perceived effectiveness and standard deviation of lower and upper half of gaming time per week participants, as well as difference between the lower and upper half indicated with t value, degrees of freedom (df), and *P* value.

| Condition | Mean perceived effectiveness (SD^a^) | | *t* value | df | *P* value |
| --- | --- | --- | --- | --- | --- |
|  |  |  |  |  |  |
|  | G1^b^ | G2^c^ |  |  |  |
| Circle | 4.06 (0.54) | 4.02 (0.61) | 0.29 | 73.06 | .77 |
| Breeze | 4.10 (0.42) | 4.09 (0.58) | 0.16 | 69.24 | .87 |

^a^SD: standard deviation.

^b^G1: participants lower half (median) of gaming time per week.

^c^G2: participants upper half (median) of gaming time per week.

**Table 4.** Moderator analysis of gaming time per week on the relationship of perceived effectiveness to condition (Breeze or Circle).

| Perceived effectiveness | Estimate | Std. Error | *t*_(152)_ | *P* value |
| --- | --- | --- | --- | --- |
|  |  |  |  |  |
| Intercept | 4.06 | 0.09 | 46.73 | <.001 |
| Condition^a^ | 0.05 | 0.12 | 0.4 | 0.69 |
| Gaming G2^b^ | -0.04 | 0.12 | -0.31 | 0.76 |
| Condition^a^:Gaming G2^b^ | 0.02 | 0.17 | 0.11 | 0.91 |
| Adjusted R^2^= -0.01595, F_(3, 152)_= 0.189, *P value=* 0.9038 | | | | |

^a^Conditon: Breeze or standard breathing training (Circle).

^b^Gaming G2: participants upper half (median) of gaming time per week.

### Intention to engage

**Table 5.** Mean intention to engage and standard deviation of lower and upper half of gaming time per week participants, as well as difference between the lower and upper half indicated with *t* value, degrees of freedom (df) and *P* value.

| Condition | Mean intention to engage (SD^a^) | | *t* value | df | *P* value |
| --- | --- | --- | --- | --- | --- |
|  |  |  |  |  |  |
|  | G1^b^ | G2^c^ |  |  |  |
| Circle | 3.36 (1.16) | 3.47 (1.01) | -0.46 | 74.05 | .64 |
| Breeze | 4.48 (0.96) | 4.62 (1.07) | -0.61 | 75.72 | .54 |

^a^SD: standard deviation.

^b^G1: participants lower half (median) of gaming time per week.

^c^G2: participants upper half (median) of gaming time per week.

**Table 6.** Moderator analysis of gaming time per week on the relationship of intention to engage to condition (Breeze or Circle).

| Intention to engage | Estimate | Std. Error | *t*_(152)_ | *P* value |
| --- | --- | --- | --- | --- |
|  |  |  |  |  |
| Intercept | 3.36 | 0.17 | 19.97 | <.001 |
| Condition^a^ | 0.12 | 0.24 | 0.49 | 0.62 |
| Gaming G2^b^ | 0.11 | 0.24 | 0.48 | 0.62 |
| Condition^a^:Gaming G2^b^ | 0.03 | 0.34 | 0.08 | 0.94 |
| Adjusted R^2^= -0.01196, F_(3, 152)_= 0.3894, *P value=* 0.7608 | | | |  |

^a^Conditon: Breeze or standard breathing training (Circle).

^b^Gaming G2: participants upper half (median) of gaming time per week.

## Meditation Experience

### Intrinsic Experiential Value

**Table 7.** Mean intrinsic value and standard deviation of meditation experts and no meditation experts (meditation expert: participants answering, "agree" or "strongly agree" of being experienced in meditation; no meditation expert: participants answering, " neither agree nor disagree", "disagree", or "strongly disagree" of being experienced in meditation), as well as difference between those indicated with *t* value, degrees of freedom (df) and *P* value.

| Condition | Mean intrinsic value (SD^a^) | | *t* value | df | *P* value |
| --- | --- | --- | --- | --- | --- |
|  |  |  |  |  |  |
|  | Meditation expert^b^ | No medidation expert^c^ |  |  |  |
| Circle | 3.16 (1.01) | 3.37 (1.1) | -0.03 | 19.87 | .98 |
| Breeze | 3.79 (0.89) | 3.49 (1.03) | -2.3 | 20.24 | .03 |

^a^sd: standard deviation.

^b^meditation expert: participants answering "agree" and "strongly agree" of being experienced in meditation.

^c^no meditation expert: participants answering, "neither agree nor disagree", "disagree", and "strongly disagree" of being experienced in meditation.

**Table 8.** Moderator analysis of meditation experience (expert or no expert) on the relationship of intrinsic value to condition (Breeze or Circle).

| Intrinsic value | Estimate | Std. Error | *t*_(152)_ | *P* value |
| --- | --- | --- | --- | --- |
|  |  |  |  |  |
| Intercept | 3.12 | 0.08 | 37.94 | <.001 |
| Condition^a^ | 0.29 | 0.12 | 2.50 | .01 |
| Meditation Expert^b^ | 0.01 | 0.19 | 0.03 | .98 |
| Condition^a^:Meditation Expert^b^ | 0.35 | 0.27 | 1.30 | .20 |
| Adjusted R^2^= 0.07086, F_(3, 152)_= 4.94, *P* value*=* 0.002659 | | | | |

^a^Conditon: Breeze or standard breathing training (Circle).

^b^Meditation expert: participants answering "agree" and "strongly agree" of being experienced in meditation.

### Perceived Effectiveness

**Table 9.** Mean perceived effectiveness and standard deviation of meditation experts and no meditation experts (meditation expert: participants answering "agree" or "strongly agree" of being experienced in meditation; no meditation expert: participants answering, "neither agree nor disagree", "disagree", or "strongly disagree" of being experienced in meditation), as well as difference between those indicated with *t* value, degrees of freedom (df) and *P* value.

| Condition | Mean perceived effectivness (SD^a^) | | *t* value | df | *P* value |
| --- | --- | --- | --- | --- | --- |
|  |  |  |  |  |  |
|  | Meditation expert^b^ | No meditation expert^c^ |  |  |  |
| Circle | 4.17 (0.46) | 4.01 (0.59) | -1.11 | 23.88 | .28 |
| Breeze | 4.11 (0.76) | 4.09 (0.44) | -0.07 | 14.94 | .94 |

^a^SD: standard deviation.

^b^Meditation expert: participants answering "agree" and "strongly agree" of being experienced in meditation.

^c^No meditation expert: participants answering, "neither agree nor disagree", "disagree" and "strongly disagree" of being experienced in meditation.

**Table 10.** Moderator analysis of meditation experience (expert or no expert) on the relationship of perceived effectiveness to condition (Breeze or Circle).

| Perceived effectiveness | Estimate | Std. Error | *t*_(152)_ | *P* value |
| --- | --- | --- | --- | --- |
|  |  |  |  |  |
| Intercept | 4.01 | 0.07 | 58.87 | <.001 |
| Condition^a^ | 0.08 | 0.10 | 0.88 | 0.38 |
| Meditation Expert^b^ | 0.16 | 0.16 | 0.99 | 0.32 |
| Condition^a^:Meditation Expert^b^ | -0.14 | 0.23 | -0.64 | 0.52 |
| Adjusted R^2^= -0.01011, F_(3, 152)_= 0.4827, *P* value*=* 0.6948 | | | | |

^a^Conditon: Breeze or standard breathing training (Circle).

^b^Meditation expert: participants answering "agree" and "strongly agree" of being experienced in meditation.

### Intention to engage

**Table 11.** Mean intention to engage and standard deviation of meditation experts and no meditation experts (meditation expert: participants answering "agree" and "strongly agree" of being experienced in meditation; no meditation expert: participants answering, "neither agree nor disagree", "disagree" and "strongly disagree" of being experienced in meditation), as well as difference between those indicated with *t* value, df and *P* value.

| Condition | Mean intention to engage (SD^a^) | | *t* value | df | *P* value |
| --- | --- | --- | --- | --- | --- |
|  |  |  |  |  |  |
|  | Meditation expert^b^ | No meditation expert^c^ |  |  |  |
| Circle | 3.64 (1.01) | 3.37 (1.1) | -0.92 | 20.44 | .37 |
| Breeze | 3.79 (0.89) | 3.49 (1.03) | -1.08 | 21.22 | .29 |

^a^SD: standard deviation.

^b^Meditation expert: participants answering "agree" and "strongly agree" of being experienced in meditation.

^c^No meditation expert: participants answering, "neither agree nor disagree", "disagree" and "strongly disagree" of being experienced in meditation.

**Table 12.** Moderator analysis of meditation experience (expert or no expert) on the relationship of intention to engage to condition (Breeze or Circle).

| Intention to engage | Estimate | Std. Error | *t*_(152)_ | *P* value |
| --- | --- | --- | --- | --- |
|  |  |  |  |  |
| Intercept | 3.37 | 0.13 | 25.53 | <.001 |
| Condition^a^ | 0.13 | 0.18 | 0.69 | 0.49 |
| Meditation Expert^b^ | 0.28 | 0.31 | 0.9 | 0.37 |
| Condition^a^:Meditation Expert^b^ | 0.02 | 0.44 | 0.04 | 0.97 |
| Adjusted R^2^= -0.004517, F_(3, 152)_= 0.7677, *P* value*=* 0.5138 | | | | |

^a^Conditon: Breeze or standard breathing training (Circle).

^b^Meditation expert: participants answering "agree" and "strongly agree" of being experienced in meditation.
